# A physarum-inspired prize-collecting steiner tree approach to identify subnetworks for drug repositioning

**DOI:** 10.1186/s12918-016-0371-3

**Published:** 2016-12-05

**Authors:** Yahui Sun, Pathima Nusrath Hameed, Karin Verspoor, Saman Halgamuge

**Affiliations:** 1 Department of Mechanical Engineering, University of Melbourne, Parkville, Melbourne, 3010 Australia; 2 Data61, Victoria Research Lab, West Melbourne, 3003 Australia; 3 Department of Computer Science, University of Ruhuna, Matara, 81000 Sri Lanka; 4 Department of Computing and Information Systems, University of Melbourne, Parkville, Melbourne, 3010 Australia; 5 Research School of Engineering, College of Engineering & Computer Science, The Australian National University, Canberra, 2601 ACT Australia

**Keywords:** Steiner tree problem, Subnetwork identification, Drug similarity network, Big data, Physarum polycephalum

## Abstract

**Background:**

Drug repositioning can reduce the time, costs and risks of drug development by identifying new therapeutic effects for known drugs. It is challenging to reposition drugs as pharmacological data is large and complex. Subnetwork identification has already been used to simplify the visualization and interpretation of biological data, but it has not been applied to drug repositioning so far. In this paper, we fill this gap by proposing a new Physarum-inspired Prize-Collecting Steiner Tree algorithm to identify subnetworks for drug repositioning.

**Results:**

Drug Similarity Networks (DSN) are generated using the chemical, therapeutic, protein, and phenotype features of drugs. In DSNs, vertex prizes and edge costs represent the similarities and dissimilarities between drugs respectively, and terminals represent drugs in the cardiovascular class, as defined in the Anatomical Therapeutic Chemical classification system. A new Physarum-inspired Prize-Collecting Steiner Tree algorithm is proposed in this paper to identify subnetworks. We apply both the proposed algorithm and the widely-used GW algorithm to identify subnetworks in our 18 generated DSNs. In these DSNs, our proposed algorithm identifies subnetworks with an average Rand Index of 81.1%, while the GW algorithm can only identify subnetworks with an average Rand Index of 64.1%. We select 9 subnetworks with high Rand Index to find drug repositioning opportunities. 10 frequently occurring drugs in these subnetworks are identified as candidates to be repositioned for cardiovascular diseases.

**Conclusions:**

We find evidence to support previous discoveries that nitroglycerin, theophylline and acarbose may be able to be repositioned for cardiovascular diseases. Moreover, we identify seven previously unknown drug candidates that also may interact with the biological cardiovascular system. These discoveries show our proposed Prize-Collecting Steiner Tree approach as a promising strategy for drug repositioning.

**Electronic supplementary material:**

The online version of this article (doi:10.1186/s12918-016-0371-3) contains supplementary material, which is available to authorized users.

## Background

Drug repositioning aims to identify new therapeutic effects for known drugs. By repositioning known drugs, drug development time, costs and risks can be reduced significantly [1–3]. There are mainly two challenges to reposition drugs. First, pharmacological data is usually big and difficult to analyze [4, 5]. Second, pharmacological data is highly complex and involves various drug characteristics, including their chemical structures, molecular targets and induced gene expression signatures [6].

Existing drug repositioning methods can be divided into three categories; data-driven methods [1–3, 6], text-mining methods [7, 8], and network-based methods [3, 9–11]. The data-driven methods reposition drugs by analyzing pharmacological data using statistical and machine learning concepts such as statistical estimations, classification and clustering [1, 6, 10]. Because of the overlapping nature of pharmacological data [3, 11], the evaluation process of the data-driven methods is complicated [11]. On the other hand, text mining methods use efficient text analytics and semantic inference approaches to reposition drugs [7, 8], but their application is limited by the availability of relevant biomedical publications and reports. Network-based methods are emerging methods that use networks to represent pharmacological data [10]. These methods typically reposition drugs by identifying drug candidates in multiple decomposed subnetworks [10–12]. Even though multiple therapeutic effects are expected to be found, it requires a long time to analyze these multiple decomposed subnetworks.

Subnetwork identification is a technique to identify a single small-scale subnetwork from a large-scale network. It differs from previous network-based methods in that we only need to analyze a single identified subnetwork. This method has already been proven to be efficient to simplify the visualization and interpretation of protein-protein interaction networks [13–16], protein-DNA interaction networks [17], gene-regulatory networks [18] and metabolic networks [19]. However, to our knowledge, no one has applied subnetwork identification to pharmacological networks so far. This paper will fill this gap by exploring the application of subnetwork identification to drug repositioning for the first time.

The Prize-Collecting Steiner Tree (PCST) approach is gaining traction in subnetwork identification, but has not been tried with pharmacological data yet. Existing methods are slow and non-deterministic, chance based. This method is heuristic, i.e. it is not an exact solution, but it is deterministic. The definition of Prize-Collecting Steiner Tree Problem (PCSTP) is given as follows: let *G*=(*V*,*E*,*p*,*c*) be a connected, undirected graph, where *V* is the set of vertices, *E* is the set of edges, *p* is a function which maps each vertex in *V* to a non-negative number called the prize, and *c* is a function which maps each edge in *E* to a positive number called the cost. Let *T* be a subset of *V* called terminals. The aim of PCSTP is to find a connected subgraph *G* ^′^=(*V* ^′^,*E* ^′^),*V* ^′^⊆*V*,*E* ^′^⊆*E* which contains all the terminals while minimizing the objective function $c(G')=\sum _{e \in E'}{c(e)}-\sum _{v \in V'}{p(v)}$, and the optimal solution of PCSTP is called Steiner Minimum Tree (SMT) in *G* for *T*.

The algorithms for PCSTP can be divided into two groups: exact algorithms and heuristic algorithms. Exact algorithms can find SMT, but are slow in large graphs [20]. On the contrary, heuristic algorithms can find solutions faster, but they may only find close approximations to SMT [21]. The Drug Similarity Networks (DSN) we used in this paper are large graphs with 548 vertices and thousands of edges. Thus, it is necessary for us to use heuristic algorithms in DSNs. Many heuristic algorithms have been proposed to solve PCSTP; the GW algorithm (named for Michel X. Goemans and David P. Williamson) is the most popular one [22–25]. However, in our simulations we observe that GW algorithm does not perform well in DSNs. Physarum-inspired algorithms are emerging heuristic algorithms that have already been used to solve PCSTP [26]. In this paper, we propose a new Physarum-inspired algorithm called Physarum-inspired Subnetwork Identification Algorithm (PSIA) to identify subnetworks in DSNs. Our proposed PSIA outperforms the popular GW algorithm by identifying more suitable subnetworks for drug repositioning. Furthermore, by analyzing the identified subnetworks, we find evidence to support previous discoveries that some drugs could be repositioned for cardiovascular diseases. These discoveries show that our proposed Prize-Collecting Steiner Tree approach is effective and efficient to reposition drugs.

## Methods

### Generation of drug similarity networks

We build Drug Similarity Networks (DSNs) to represent the similarities between drugs. There are several pharmacological databases at present, such as PharmGKB [27], DrugBank [5, 28], SIDER [29], etc. We generate DSNs using the data following the work of Zhang et al. [30], which includes data from DrugBank and SIDER. Similarities between drugs are quantified in DSNs based on their chemical, therapeutic, protein and phenotype features. There are 881 chemical features, 719 therapeutic features, 775 protein features, and 1385 phenotype features considered for each drug. Therefore, 3760 (881+719+775+1385) features in total are considered for each drug.

The DSNs we generated have five components:

- **vertex**: Each vertex represents a drug. There are 548 drugs included in each of our generated DSNs [30]. Each drug is associated with a 1×3760 feature vector where binary numbers represent the presence or absence of each individual feature that we consider. Note that, binary numbers have already been widely used to describe drug features [6, 30, 31].
- **edge**: Each edge represents the association between two drugs.
- **terminal**: Each terminal represents a vertex which must be contained in the identified subnetworks of DSNs. In each DSN, the terminal set represents a cardiovascular subclass of drugs in the Anatomical Therapeutic Chemical (ATC) classification system [32]. ATC is used for the classification of active ingredients of drugs according to the organ or system on which they act and their therapeutic, pharmacological and chemical properties. There are 9 subclasses in the cardiovascular class (C); cardiac therapy (C01), antihypertensives (C02), diuretics (C03), peripheral vasodilators (C04), vasoprotectives (C05), beta blocking agents (C07), calcium channel blockers (C08), agents acting on the renin-angiotensin system (C09), and lipid modifying agents (C10). There are 104 drugs in total in these subclasses. (Notably, there is no C06 in the ATC classification system).
- **edge cost**: Each edge cost represents the quantified dissimilarity between two drugs. The bigger the edge cost is, the more dissimilar the two drugs are. The edge cost is calculated using the Jaccard coefficient, as shown in the formula below.
  1 $$ \begin{aligned} c_{ij}=1-\frac{\sum_{k=1}^{n}{\upsilon_{i}(k) \cap \upsilon_{j}(k)}}{\sum_{k=1}^{n}{\upsilon_{i}(k) \cup \upsilon_{j}(k)}}  \end{aligned}  $$
  where *i* and *j* are indexes of two different drugs, *c*_*ij*_ is the cost of edge (*i*,*j*), *n* is the total number of features considered for each drug, which is 3760, and *υ*_*i*_ is the feature vector of drug *i*.
- **vertex prize**: A prize is associated with each vertex to signify the similarity between the drug represented by this vertex and all the drugs represented by terminals. The vertex prize is calculated using the following equation.
  2 $$ p_{i}=\frac{\sum_{j \in T, j \neq i}{\frac{1}{1+c_{ij}}}}{|T|}   $$
  where *p*_*i*_ is the prize of vertex *i*, *T* is the set of terminals, and |*T*| is the total number of terminals.

The objective of PCSTP is to minimize the net-cost of edge costs and vertex prizes. Thus, the subnetwork identified using the PCST approach tends to include edges with small costs and vertices with big prizes. In our generated DSNs, edges with small costs connect drugs with big similarities, and vertices with big prizes represent drugs that are similar to the drugs represented by terminals. Hence, a subnetwork of DSN that includes drugs similar to the drugs represented by terminals is expected to be identified using the PCST approach.

Complete graphs with different sets of terminals can be generated using the five graph components defined above. Since the sets of vertices are identical, the sets of edge costs are also the same in different complete graphs. However, the sets of vertex prizes are different as the sets of terminals are different in different complete graphs. PCSTP algorithms perform better in sparse graphs than in complete graphs [22]. Therefore, we propose two sparse graph generation algorithms to prune the complete graphs to produce sparse graphs for DSNs.

**Fig. 1:**
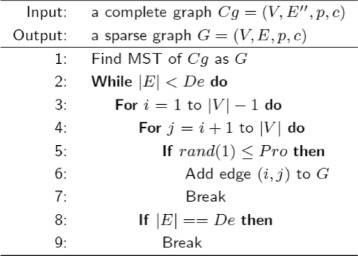
The first proposed sparse graph generation algorithm

In our first proposed algorithm, the Minimum Spanning Tree (MST) of the complete graphs is found using Prim’s algorithm [33]. Then, edges are added probabilistically to MST until the total number of edges is increased to the desired number. This algorithm is outlined in Fig. 1, in which |*E*| is the number of edges in the sparse graph, |*V*| is the number of vertices, *De* is the desired number of edges in the sparse graph, *Pro* is the probability of adding edges to MST.

**Fig. 2:**
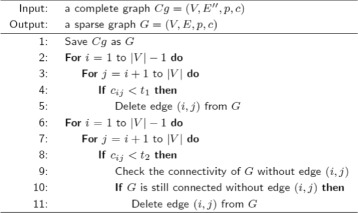
The second proposed sparse graph generation algorithm

Our first proposed algorithm generates a sparse graph by adding edges to the MST of a complete graph. While our second proposed algorithm generates a sparse graph by deleting edges from a complete graph. The challenge of generating a sparse graph by deleting edges is to delete as many edges as possible while maintaining the graph connectivity. The graph connectivity can be checked using Tarjan’s algorithm, which has the complexity of *O*(|*V*|+|*E*|) [34]. It takes a long time to generate a sparse graph if the connectivity is checked every time after an edge is deleted. In our second proposed algorithm, two threshold values, *t*_1_ and *t*_2_, are used to delete edges in two steps. In the first step, all the edges which have a cost below *t*_1_ are deleted from the complete graph. In the second step, all the edges which have a cost below *t*_2_ are deleted from the graph when deleting the edge will not make the graph disconnected. Set *t*_1_<*t*_2_, and make sure *t*_1_ is small enough to maintain the graph connectivity. The purpose of deleting edges in two steps is to make the algorithm fast by only checking the graph connectivity in the second step. Our second proposed algorithm is outlined in Fig. 2.

**Fig. 3:**
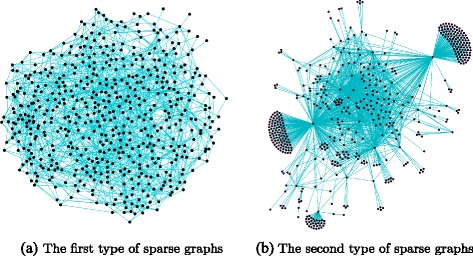
Visualization of two types of sparse graphs. **a** shows the first type of sparse graphs, which are generated using the first proposed algorithm. **b** shows the second type of sparse graphs, which are generated using the second proposed algorithm

Two sparse graphs can be generated from each complete graph using the two algorithms proposed above. These two algorithms generate sparse graphs by only considering the edge costs. Since the sets of edge costs are the same in different complete graphs, the sparse graphs generated using the same proposed algorithm will have the same set of edges. Therefore, sparse graphs with two different sets of edges are generated using the two proposed algorithms, and these two types of sparse graphs are visualized in Fig. 3, in which Fig. 3 a visualizes the sparse graphs generated using the first proposed algorithm, and there are 548 vertices and 1500 edges in each of them, Fig. 3 b visualizes the sparse graphs generated using the second proposed algorithm, and there are 548 vertices and 1391 edges in each of them.

**Fig. 4:**
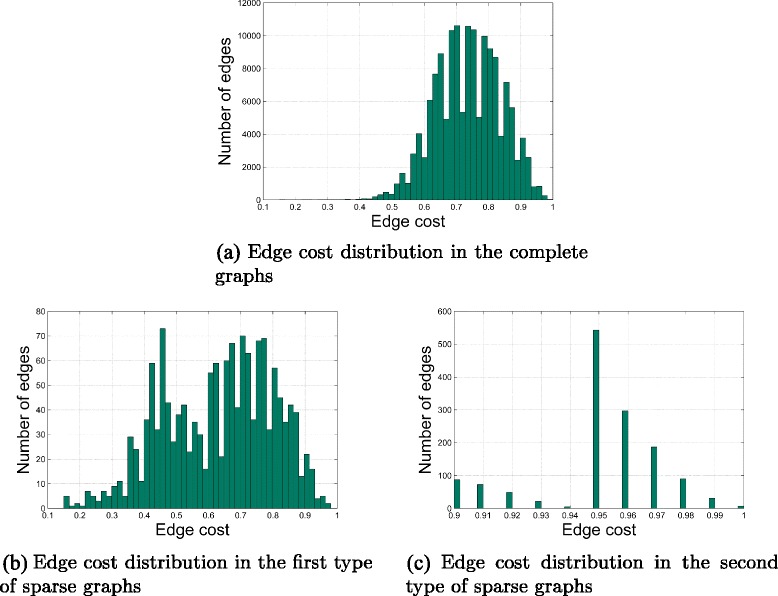
The distributions of edge costs in the complete graphs and the sparse graphs. **a** shows the distribution of edge costs in the complete graphs. **b** shows the distribution of edge costs in the first type of sparse graphs, which are generated using the first proposed algorithm. **c** shows the distribution of edge costs in the second type of sparse graphs, which are generated using the second proposed algorithm

The distributions of edge costs in the complete graphs and two types of sparse graphs are shown in Fig. 4. It can be seen from Fig. 4 a that most edges in the complete graphs have costs between 0.5 and 0.9. It can be seen from Fig. 4 b that most edges in the first type of sparse graphs also have costs between 0.5 and 0.9. The reason why the complete graphs and the first type of sparse graphs have similar distributions of edge costs is that, in the first proposed algorithm, edges are randomly added to the MST of the complete graphs without considering their costs. However, it can be seen from Fig. 4 c that most edges in the second type of sparse graphs have costs between 0.9 and 1. It is because *t*_1_ and *t*_2_ are set respectively to be 0.9 and 0.95 in the second proposed algorithm, and all the edges which have a cost below 0.9 have been deleted. In the computational trials, it takes the second proposed algorithm 29.24 s to generate a sparse graph when *t*_1_=0.9 and *t*_2_=0.95. In contrast, it takes the second proposed algorithm 7870.69 seconds to generate a sparse graph when *t*_1_=0.5 and *t*_2_=0.95. Moreover, the graph becomes disconnected when *t*_1_=0.95. Therefore, the computational trials prove that a big *t*_1_ makes the second proposed algorithm fast, but at the risk of ruining the graph connectivity, and a big *t*_2_ makes the graph sparse, but at the cost of long running time.

DSNs in sparse graphs are generated using the two proposed algorithms. Because no vertex has been deleted in any of these sparse graphs, subnetworks containing similar drugs can be identified in the sparse graphs generated by both proposed algorithms. Nevertheless, in our simulations we find that PCSTP algorithms have better performances in DSNs generated using the second proposed algorithm than in DSNs generated using the first proposed algorithm.

### The proposed physarum-inspired subnetwork identification algorithm

Physarum polycephalum is a large amoeboid organism that has displayed many intelligent behaviors [35–37]. The Physarum-inspired Subnetwork Identification Algorithm (PSIA) is proposed in this paper to identify subnetworks in DSNs. The proposal of PSIA is inspired by the Lowest-cost Network Physarum Optimization algorithm (LNPO) [26]. LNPO is designed to find PCSTP solutions as close to SMT as possible. There are two iteration processes in LNPO, the inner iteration process and the outer iteration process. A feasible PCSTP solution can be found in each inner iteration process. The outer iteration process is used to find multiple solutions and choose the solution which is closest to SMT as the final solution. However, SMT or close approximations to SMT may not be suitable for drug repositioning. There is no need to use the outer iteration process in PSIA. Thus, only the inner iteration process is included in PSIA. Moreover, the subnetwork identified in the inner iteration process may not be a tree. Hence, a post-processing technique is used in PSIA to ensure that the identified subnetwork is a tree.

In our proposed PSIA algorithm, a single terminal is chosen probabilistically to be the sink node, and all the other terminals will become source nodes. Let *l*(*i*) be the total cost of edges linked to terminal *i*. Name the terminals in such a way that *l*(1)≤*l*(2)≤⋯≤*l*(|*T*|), where |*T*| is the number of terminals. Then, the probability of choosing terminal *i* as the sink node can be obtained by

3 $$\begin{array}{@{}rcl@{}} P(i)= \frac{l(|T|-i+1)}{\sum_{j=1}^{|T|} l(j)} \end{array} $$

There is flux flowing through each edge, and the flux *Q*_*ij*_ in edge (*i*,*j*) is given by

4 $$\begin{array}{@{}rcl@{}} Q_{ij} = \frac{D_{ij}}{C_{ij}} \left({Pr}_{i} - {Pr}_{j}\right) \end{array} $$

5 $$\begin{array}{@{}rcl@{}} C_{ij}=c_{ij}-\frac{p_{i}}{d_{i}}-\frac{p_{j}}{d_{j}}+2N \end{array} $$

where *D*_*ij*_ is the edge conductivity, *C*_*ij*_ is a net-cost for edge (*i*,*j*), *P* *r*_*i*_ and *P* *r*_*j*_ are pressures at vertex *i* and *j*, *c*_*ij*_ is the cost of edge (*i*,*j*), *p*_*i*_ and *p*_*j*_ are the prizes of vertex *i* and *j*, *d*_*i*_ and *d*_*j*_ are the degrees of vertex *i* and *j*, and *N*=*m* *a* *x*(*p*_*k*_),*k*⊆*V*.

The flux flows into the network from each source node, and the flux flows out of the network from the single sink node. By considering the conservation law of flux at each vertex, the network Poisson equation is described below.

6 $$\begin{array}{@{}rcl@{}} \sum_{i \in V(j)} \frac{D_{ij}}{C_{ij}} ({Pr}_{i} - {Pr}_{j}) =\left\{ \begin{array}{cl} -I_{0}, & j=source\\ +(|T|-1)I_{0}, & j=sink\\ 0, & otherwise \end{array}\right. \end{array} $$

where *V*(*j*) is the set of vertices linked to vertex *j*, and *I*_0_ is the flux flowing into each source node. Let the pressure at the sink node be 0, and other pressures can be calculated by solving the network Poisson equation. In our simulations, we find that the net-costs of edges in DSNs are quite close to each other. In this case, if all the edge conductivities are the same, then the network Poisson equation may not be solvable. Thus, we give each edge conductivity a random initial value to make the network Poisson equation solvable.

**Fig. 5:**
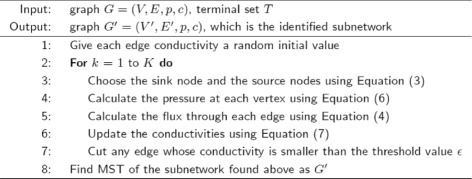
The proposed physarum-inspired subnetwork identification algorithm

After the calculation of pressures, the flux in each edge can be got. Edge conductivities will be updated using the conductivity update equation below.

7 $$\begin{array}{@{}rcl@{}} D_{ij}(k+1)=D_{ij}(k)+\alpha|Q_{ij}(k)|-\mu D_{ij}(k) \end{array} $$

where *k* is the number of conductivity update times, *α* and *μ* are two constants. Edges with conductivities smaller than the threshold value *ε* will be cut from the graph. We iteratively update the edge conductivities and cut edges for *K* times to find a subnetwork. However, this subnetwork may not be a tree. Thus, MST of this subnetwork is found to be the final identified subnetwork. The proposed PSIA is outlined in Fig. 5 (the MATLAB coding of PSIA is publicly available at https://github.com/YahuiSun/PSIA-to-identify-subnetworks).

Because the sink node is chosen probabilistically in PSIA, different subnetworks can be identified in a single DSN by employing PSIA for multiple times. To reposition drugs, we employ PSIA for multiple times in each DSN to identify multiple subnetworks. Then, we select the most suitable subnetwork from them for drug repositioning.

### GW algorithm

Besides the proposed PSIA, we also use the popular GW algorithm to identify subnetworks in DSNs. GW algorithm was proposed by Michel X. Goemans and David P. Williamson [22], and it is widely used to solve PCSTP [23–25]. However, GW algorithm is designed to solve PCSTP instances with a single terminal, which is called the root. While in DSNs, there are multiple terminals. In this paper, we apply GW algorithm to DSNs by randomly choosing a single terminal to be the root and give other terminals big prizes.

We first choose a single terminal to be the root. Then, we give each of the other terminals a big prize *M*, and $M > \sum _{(i,j) \in E} c_{ij}$. This big prize ensures that all the terminals will be included in the subnetwork identified by GW algorithm.

To identify a subnetwork, we initially set each vertex as a component. Each component has a surplus (initially the vertex prize). A component is active when its surplus is bigger than 0. However, the root component will always be inactive. In addition, each edge has a deficit (initially the edge cost), and an edge is active when it is not connecting two vertices in the same component.

Setting a constant *Δ*, we iteratively do this: the surplus of all active components are reduced by *Δ*, the deficit of any active edge adjacent to a single active component is reduced by *Δ*, and the deficit of any active edge adjacent to two active components is reduced by 2*Δ*. After the update of surpluses and deficits, we check that: if an edge’s deficit is not above 0, we merge the two components linked by this edge and give the new merged component the sum of surpluses of the two components being merged; if a component’s surplus is not above 0, we deactivate this component. The iteration will end until there is no active component disconnected with the root component.

After the iteration, the vertices and the edges in the root component will be a tree. Then, we delete some vertices and edges by strong pruning the tree. The strong pruning idea was proposed by Johnson et al. in 2000 [25]. In the general GW algorithm, MST of the strong pruned tree is recommended to be found to increase the total net-prize of the identified subnetwork. However, in this paper, the aim of identifying subnetworks is to identify drug candidates, which are vertices in DSNs. Therefore, it is not necessary to find MST of the strong pruned tree, and we can directly use the strong pruned tree as the identified subnetwork for drug repositioning. The MATLAB coding of GW algorithm is publicly available at https://github.com/YahuiSun/GW-to-identify-subnetworks.

### Subnetwork evaluation for drug repositioning

As described above, we select each of the 9 cardiovascular subclasses individually as the terminal set, and all the other drugs in the DSN are considered as non-terminal vertices. We then apply two sparse graph generation algorithms to generate two sparse graphs for each cardiovascular subclass, resulting in 18 DSNs. We name each DSN as *D*_*i*_*a* or *D*_*i*_*b*, in which *i* represents the origin of the terminal set (subclass C01, C02, C03, C04, C05, C07, C08, C09, or C10), *a* or *b* represents the first or the second sparse graph generation algorithm that is used to generate that particular DSN.

Both PSIA and GW algorithm have been applied to each of the 18 DSNs to identify subnetworks. PSIA can identify multiple subnetworks in each DSN, while GW algorithm can only identify a single subetwork in each DSN. Each identified subnetwork contains all the terminals and may also contain some non-terminal vertices. In DSNs, the drugs represented by terminals are in a certain cardiovascular subclass, while the drugs represented by non-terminal vertices may or may not be in the other cardiovascular subclasses. The aim of subnetwork identification is to reposition drugs for cardiovascular diseases. Drugs in the cardiovascular class are closely related to cardiovascular diseases. Moreover, the identified subnetwork is supposed to contain drugs that are closely related to each other. Therefore, a subnetwork that is suitable for drug repositioning for cardiovascular diseases may contain a high percentage of drugs that are in the cardiovascular class and a low percentage of drugs that are not in the cardiovascular class. Hence, we propose Rand Index (RI) [38] as the metric to evaluate the identified subnetworks, and it is defined as

8 $$  \text{RI}=\frac{I_{c} + N_{nc}}{|V|-|T|} \times 100\%  $$

where *I*_*c*_ is the number of non-terminal vertices that represent drugs that are in both the identified subnetwork and the cardiovascular class (C; including drugs in all 9 cardiovascular subclasses), *N*_*nc*_ is the number of vertices that represent drugs that are neither in the identified subnetwork nor in the cardiovascular class, |*V*| is the number of vertices in DSN (|*V*|=548 in this paper), |*T*| is the number of terminals in DSN. Notably, our computational trials show that identifying true positives (*I*_*c*_) and true negatives (*N*_*nc*_) are both important to subnetwork identification for drug repositioning.

We evaluate all the subnetworks identified by PSIA and GW algorithm. Then, we select the subnetworks with high RI as the suitable subnetworks for drug repositioning. Most drugs in these selected subnetworks have already been classified into the cardiovascular class. However, there may still be drugs in these selected subnetworks that have not been classified into the cardiovascular class yet. We consider the ‘not-classified-yet’ drugs that have frequently occurred in these selected subnetworks as candidates for drug repositioning.

## Results

There are two groups of DSNs generated in this paper. Each group contains 9 DSNs that are generated using 9 cardiovascular subclasses (C01, C02, C03, C04, C05, C07, C08, C09, C10). The DSNs in the first group (*D*_01_*a* to *D*_10_*a*) are generated using the first proposed sparse graph generation algorithm (Fig. 1), while the DSNs in the second group (*D*_01_*b* to *D*_10_*b*) are generated using the second proposed sparse graph generation algorithm (Fig. 2). These DSNs are publicly available at https://github.com/YahuiSun/Drug-Similarity-Network.

Both PSIA and GW algorithm are used to identify subnetworks in two groups of DSNs. Since PSIA can identify multiple subnetworks in a single DSN, we employ PSIA for three times in each DSN to identify three subnetworks.

In each DSN, the subnetwork with the highest RI identified by PSIA is selected to compare with the subnetwork identified by GW algorithm. The comparison results are shown in Tables 1 and 2, in which ID is the name of DSN, |*V*|, |*E*|, |*T*| are the numbers of vertices, edges, terminals in each DSN, T-Origin is the origin of the terminal set in each DSN, |*V* ^′^| and |*E* ^′^| are the numbers of vertices and edges in each identified subnetwork.

**Table 1:** Subnetwork identification results in drug similarity network: *D*_01_*a* to *D*_10_*a*

DSN					Identified subnetwork				
ID	|*V*|	|*E*|	|*T*|	T-Origin	Algorithm	|V’ |	|E’ |	*I*_*c*_	Rand Index
D_01_a	548	1500	22	C01	PSIA	60	59	**7**	**79.8**
					GW	354	353	53	41.4
D_02_a	548	1500	12	C02	PSIA	37	36	**10**	**81.9**
					GW	339	338	62	45.0
D_03_a	548	1500	13	C03	PSIA	35	34	**4**	**80.4**
					GW	330	329	61	46.5
D_04_a	548	1500	4	C04	PSIA	9	8	**1**	**81.1**
					GW	322	321	66	47.4
D_05_a	548	1500	9	C05	PSIA	25	24	**4**	**80.9**
					GW	281	280	52	51.2
D_07_a	548	1500	15	C07	PSIA	25	24	**1**	**81.8**
					GW	301	300	55	50.3
D_08_a	548	1500	8	C08	PSIA	23	22	**2**	**80.2**
					GW	320	319	63	47.8
D_09_a	548	1500	16	C09	PSIA	29	28	**4**	**82.5**
					GW	322	321	56	47.0
D_10_a	548	1500	8	C10	PSIA	18	17	**1**	**80.7**
					GW	354	353	66	42.6

The highlighted numbers indicate the higher Rand Index and the corresponding *I*_*c*_ in each instance

**Table 2:** Subnetwork identification results in Drug Similarity Network: *D*_01_*b* to *D*_10_*b*

DSN					Identified subnetwork				
ID	|*V*|	|*E*|	|*T*|	T-Origin	Algorithm	|V’ |	|E’ |	*I*_*c*_	Rand Index
*D*_01_*b*	548	1391	22	C01	PSIA	41	40	2	81.6
					**GW**	**32**	**31**	**1**	**82.9**
*D*_02_*b*	548	1391	12	C02	**PSIA**	**22**	**21**	**2**	**81.7**
					GW	25	24	1	80.8
*D*_03_*b*	548	1391	13	C03	**PSIA**	**18**	**17**	**2**	**82.8**
					GW	20	19	1	82.1
*D*_04_*b*	548	1391	4	C04	**PSIA**	**9**	**8**	**2**	**81.4**
					GW	10	9	1	80.9
*D*_05_*b*	548	1391	9	C05	**PSIA**	**12**	**11**	**1**	**82.2**
					GW	17	16	1	81.3
*D*_07_*b*	548	1391	15	C07	**PSIA**	**23**	**22**	**2**	**82.6**
					GW	24	23	1	82.0
*D*_08_*b*	548	1391	8	C08	**PSIA**	**19**	**18**	**1**	**80.6**
					GW	19	18	0	80.2
*D*_09_*b*	548	1391	16	C09	**PSIA**	**22**	**21**	**1**	**82.7**
					GW	26	25	1	82.0
*D*_10_*b*	548	1391	8	C10	PSIA	54	53	5	75.6
					**GW**	**14**	**13**	**1**	**81.5**

The highlighted numbers indicate the higher Rand Index and the corresponding *I*_*c*_ in each instance

The identified subnetwork with a higher RI in each DSN has been highlighted in Tables 1 and 2. It can be seen that every highlighted subnetwork has a smaller number of vertices than the other subnetwork in the same DSN. Thus, we observe that

**Observation 1:** In each DSN, the identified subnetwork which has a higher RI is generally smaller than the other identified subnetwork.

It is preferable to choose small subnetworks than large subnetworks for drug repositioning as analysis can be done more efficiently in small subnetworks. Most drugs included in our generated DSNs are not in the cardiovascular class. Hence, it is important for subnetworks to identify true negatives (*N*_*nc*_ in Eq. (8)), and then avoid false positives (drugs that are not in the cardiovascular class). One counter-example is that the subnetworks identified by GW algorithm in *D*_01_*a* to *D*_10_*a* contain many false positives, and thus are large and not suitable for drug repositioning.

In *D*_01_*a* to *D*_10_*a*, all the highlighted subnetworks are identified by PSIA. In *D*_01_*b* to *D*_10_*b*, 7 out of 9 highlighted subnetworks are identified by PSIA. In 18 DSNs, the average RI of the subnetworks identified by PSIA is 81.1%, while the average RI of the subnetworks identified by GW algorithm is 64.1%. Therefore, the conclusion below can be made.

**Conclusion 1:** In our generated DSNs, PSIA generally outperforms GW algorithm in identifying subnetworks for drug repositioning.

*D*_01_*a* to *D*_10_*a* are generated using the first proposed sparse graph generation algorithm (Fig. 1), while *D*_01_*b* to *D*_10_*b* are generated using the second proposed sparse graph generation algorithm (Fig. 2). 8 out of 9 highlighted subnetworks in *D*_01_*b* to *D*_10_*b* (except *D*_02_*b*) have higher RI than the corresponding highlighted subnetworks in *D*_01_*a* to *D*_10_*a* (two DSNs corresponds to each other when they use the same cardiovascular subclass as the terminal set; see Tables 1 and 2). Hence, the conclusion below can be made.

**Conclusion 2:** The second proposed sparse graph generation algorithm is more suitable than the first proposed sparse graph generation algorithm for DSN generation.

**Fig. 6:**
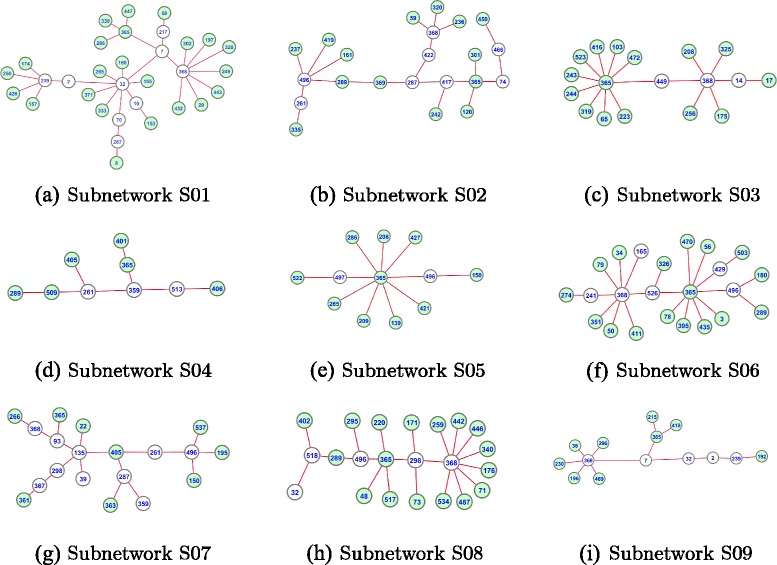
Visualization of the highlighted subnetworks in *D*_01_*b* to *D*_10_*b*. S01-S09 are IDs of the highlighted subnetworks in *D*_01_*b* to *D*_10_*b*. The numbers in the visualized subnetworks represent the indexes of drugs. The *green-color vertices* represent drugs that are in the cardiovascular class. The *white-color vertices* represent drugs that are not in the cardiovascular class

We select the nine highlighted subnetworks in *D*_01_*b* to *D*_10_*b* (which are generated using the second proposed sparse graph generation algorithm) for drug repositioning. These subnetworks are visualized in Fig. 6, in which S01-S09 are IDs of the highlighted subnetworks in *D*_01_*b* to *D*_10_*b*, the numbers in the visualized subnetworks represent the drug index (see drug names in Additional file 1), the green-color vertices represent drugs that are in the cardiovascular class, and the white-color vertices represent drugs that are not in the cardiovascular class. Drug candidates are selected from the frequently occurring drugs that are not in the cardiovascular class. These drug candidates are closely related to the cardiovascular system, and they could be repositioned for cardiovascular diseases.

## Discussion

Due to the long time, large costs and high risks to develop new drugs, drug repositioning is important since it finds new therapeutic effects for known drugs. In this paper, we propose subnetwork identification as a new method to reposition drugs. Because cardiovascular health contributes significantly to the burden of illness and injury in the Australian community [39], and the Prize-Collecting Steiner Tree (PCST) approach is a good way to identify subnetworks, we focus on using the PCST approach to reposition drugs for cardiovascular diseases.

To identify subnetworks for drug repositioning, we generate Drug Similarity Networks (DSN) including five components, which are vertices, vertex prizes, edges, edge costs, and terminals. The PCSTP algorithm tends to identify a subnetwork constructed by vertices with big prizes and edges with small costs. In our DSNs, the vertex prizes represent similarities between drugs, and the edge costs represent dissimilarities between drugs. Moreover, terminals represent drugs in the cardiovascular class. Therefore, a subnetwork of drugs that are closely related to the cardiovascular system is expected to be identified using the PCST approach.

18 DSNs are generated using 9 cardiovascular subclasses and 2 sparse graph generation algorithms. After generating DSNs, PCSTP algorithms are used to identify subnetworks. GW algorithm is one of the most popular PCSTP algorithm. However, GW algorithm is designed for the single-terminal (root) case, while there are multiple terminals in DSNs. Therefore, we first adapt GW algorithm for the multiple-terminal case and then use it to identify subnetworks in DSNs. Nevertheless, GW algorithm can only identify a single subnetwork in each DSN, and this subnetwork may not be suitable for drug repositioning. Hence, we propose a new PCSTP algorithm, Physarum-inspired Subnetwork Identification Algorithm (PSIA), to identify subnetworks in DSNs as well, and PSIA can identify multiple subnetworks in each DSN.

We employ both PSIA and GW algorithm in 18 DSNs. In each DSN, one subnetwork is identified by GW algorithm, and three subnetworks are identified by PSIA. Since Rand Index gives equal weight to the identification of true positives and true negatives, it can be used to select suitable subnetworks for drug repositioning. Thus, we evaluate these subnetworks using their Rand Index. Furthermore, the subnetwork identified by GW algorithm and the best subnetwork identified by PSIA are compared with each other in each DSN.

Based on the comparison results shown in Tables 1 and 2, we first observe that smaller subnetworks always have higher Rand Index than larger subnetworks in the same DSN. Then, we conclude that PSIA outperforms GW algorithm in DSNs. Moreover, we conclude that the second proposed sparse graph generation algorithm is more suitable than the first proposed sparse graph generation algorithm for DSN generation.

### Drug repositioning for cardiovascular diseases

After the evaluation of all the identified subnetworks, we select nine most suitable subnetworks to reposition drugs for cardiovascular diseases. These nine subnetworks are visualized in Fig. 6. The drugs contained in these subnetworks are supposed to be closely related to the cardiovascular system. There are 134 drugs contained in these subnetworks, in which 104 drugs are already in the cardiovascular class, while 30 drugs are not in the cardiovascular class yet. Therefore, we consider these 30 drugs as newly identified drugs for drug repositioning. These 30 drugs are listed in Table 3, in which Index is the drug index, Freq is the number of times each drug has been identified for, S01-S09 are IDs of the nine selected subnetworks.

**Table 3:** Newly identified drugs in the selected subnetworks

Index	Drug Name	Freq	S01	S02	S03	S04	S05	S06	S07	S08	S09
368	nitroglycerin	7	X	X	X			X	X	X	X
496	theophylline	5		X			X	X	X	X	
32	arsenic trioxide27	3	X							X	X
261	isocarboxazid	3		X		X			X		
287	lincomycin	3	X	X					X		
2	acarbose	2	X								X
7	adapalene	2	X								X
239	haloperidol	2	X								X
298	malathion	2							X	X	
359	neomycin	2				X			X		
10	alclometasone	1	X								
14	amcinonide	1			X						
39	azathioprine	1							X		
70	caffeine	1	X								
74	carbachol	1		X							
93	ceftazidime	1							X		
135	desflurane	1							X		
165	droperidol	1						X			
217	formoterol	1	X								
241	hexachlorophene	1						X			
367	nitrofurantoin	1							X		
417	pramipexole	1		X							
422	prednisone	1		X							
429	procyclidine	1						X			
449	repaglinide	1			X						
466	selegiline	1		X							
497	thiabendazole	1					X				
513	topiramate	1				X					
518	tranexamic acid	1								X	
526	triiodothyronine	1						X			

It can be seen from Table 3 that ten newly identified drugs have occurred more than once in the selected subnetworks, while the other 20 drugs have occurred only once in the selected subnetworks. We consider the ten drugs which have occurred more than once as candidates for drug repositioning. These ten drug candidates are nitroglycerin, theophylline, arsenic trioxide, isocarboxazid, lincomycin, acarbose, adapalene, haloperidol, malathion, and neomycin.

We believe that these ten drug candidates could be repositioned for cardiovascular diseases. Thus, we evaluate each drug candidate using published pharmacological discoveries. The existing discoveries on three candidates (nitroglycerin, theophylline and acarbose) are introduced below.

As to nitroglycerin, Koch et al. [40] found that nitroglycerin can produce a sharp fall in the cardiac filling pressures and the pulmonary arterial pressures. Moreover, the vasodilatory effects of nitroglycerin also have the potential to be used in cardiovascular therapeutics [41]. As to theophylline, Sollevi et al. [42] found that theophylline can act as an adenosine antagonist to antagonize cardiovascular responses. As to acarbose, Chiasson et al. [43] found that treating impaired glucose tolerance patients with acarbose is associated with a significant reduction in the risk of cardiovascular diseases and hypertension.

It can be seen from these discoveries that nitroglycerin, theophylline and acarbose have already been suspected for their potential therapeutic effects for cardiovascular diseases. Therefore, we provide evidences to support these previous discoveries. As to the other seven drug candidates, we believe that they also may interact with the biological cardiovascular system. These evidences have shown the effectiveness and efficiency of our proposed PCST approach for drug repositioning.

### Different types of drug similarities

In our generated DSNs, the edge cost represents the quantified dissimilarity between drugs, and the vertex prize represents the similarity between the drug represented by this vertex and all the drugs represented by terminals. There are different types of drug similarities with physical meanings, such as chemical similarity, therapeutic similarity, phenotype similarity, and similarity based on their interacting targets (such as proteins) [44].

In our generated DSNs, drug similarities are calculated using four types of drug features, which are the chemical, therapeutic, protein, and phenotype features. In this section, we generate new DSNs based on new drug similarities, and show that the initial drug similarities calculated using four types of drug features are the best drug similarities for drug repositioning.

We generate four new types of DSNs, and in each of them the drug similarities are calculated using a single type of drug features. The used drug features are chemical, therapeutic, protein, and phenotype features. We compare the standard deviations of vertex prizes and edge costs in the initial type of DSNs and four new types of DSNs.

The comparison results are demonstrated in Table 4, in which SD_VP is the average standard deviation of vertex prizes, SD_EC is the standard deviation of edge costs in the corresponding complete graphs, DSN_C is the type of DSNs where drug similarities are calculated using the chemical features, DSN_T is the type of DSNs where drug similarities are calculated using the therapeutic features, DSN_Pr is the type of DSNs where drug similarities are calculated using the protein features, DSN_Ph is the type of DSNs where drug similarities are calculated using the phenotype features, DSN_01_a/b to DSN_10_a/b are the initial type of DSNs used for drug repositioning, where drug similarities are calculated using all the four types of drug features.

**Table 4:** Standard deviations of vertex prizes and edge costs

	DSN_C	DSN_T	DSN_Pr	DSN_Ph	DSN_01_a/b to
					DSN_10_a/b
SD_VP	3.68	3.34	2.32	2.24	2.42
SD_EC	15.63	7.72	7.43	8.90	10.14

It can be seen from Table 4 that SD_VP and SD_EC of DSN_C are higher than that of other types of DSNs. It is recommended to select DSNs with high standard deviations for drug repositioning as it is hard to identify drug repositioning candidates in DSNs with low standard deviations. However, many drugs undergo complex and largely uncharacterized metabolic transformations, and the physiological effects of drugs may not be able be predicted by their chemical properties alone [45]. Therefore, it is not appropriate to only consider chemical similarities for drug repositioning. Similarly, it is not appropriate to only consider any other homogeneous drug similarity either [11]. The initial drug similarities are heterogeneous as they are calculated using multiple types of drug features. It can also be seen from Table 4 that SD_VP and SD_EC of DSN_01_a/b to DSN_10_a/b are also relatively high. Therefore, the initial heterogeneous drug similarities calculated using four types of drug features are the best drug similarities for drug repositioning.

### The running time in large drug similarity networks

We use the PCST approach to identify subnetworks for drug repositioning. The Prize-Collecting Steiner Tree Problem is NP-hard [46], which means that the time required to solve it may increase exponentially as the graph size increases. Large DSNs with thousands of vertices can be generated using the existing pharmacology data. Thus, it is necessary to ensure that we can use the PCST approach to identify subnetworks in large DSNs.

In this section, random DSNs with different sizes are generated. We employ both PSIA and GW algorithm in these DSNs using MATLAB R2014a on a computer with 16 GB RAM and the Intel(R) Core(TM) i7-4770 CPU. The running time of PSIA and GW algorithm in these DSNs is demonstrated in Table 5, in which DSN_X means a DSN with X vertices. The unit of the running time is minute.

**Table 5:** The running time of PSIA and GW algorithm in DSNs with different sizes

	DSN_100	DSN_548	DSN_1000	DSN_3000
GW	0.001 min	0.039 min	0.196 min	7.345 min
PSIA	0.036 min	0.638 min	2.102 min	19.169 min

It can be seen from Table 5 that both PSIA and GW algorithm can identify subnetworks in large DSNs with up to 3000 vertices in a reasonable time. Moreover, the running time above can be further shortened by using a low-level programming language. Thus, we can use the PCST approach to identify subnetworks in large DSNs. Notably, even though the running time of PSIA is longer than that of GW algorithm, PSIA is considered better as it can identify more suitable subnetworks for drug repositioning.

## Conclusions

Drug repositioning is important for drug development. In this paper, the subnetwork identification method is used to reposition drugs for the first time. A new Price-Collecting Steiner Tree algorithm is proposed in this paper to identify subnetworks. The popular GW algorithm is also used to compare with our proposed algorithm. Drug Similarity Networks are generated, in which vertex prizes and edge costs represent the similarities and dissimilarities between drugs respectively, and terminals represent drugs in the cardiovascular class, as defined in the Anatomical Therapeutic Chemical classification system. In the generated Drug Similarity Networks, our proposed algorithm identifies subnetworks with higher Rand Index than the popular algorithm. Furthermore, nine most suitable subnetworks are selected for drug repositioning, and ten drug candidates are identified from these subnetworks. We find evidence to support previous discoveries that nitroglycerin, theophylline and acarbose may be able to be repositioned for cardiovascular diseases. Moreover, we identify seven previously unknown drug candidates that also may interact with the biological cardiovascular system. Therefore, our proposed Prize-Collecting Steiner Tree approach is shown to be a promising strategy for drug repositioning.

## Additional file

Additional file 1 This file contains a long table of the drug indexes and names. (PDF 42 kb)
