# Supplementary material for: A physarum-inspired prize-collecting steiner tree approach to identify subnetworks for drug repositioning
Source: BMC Syst Biol. 2016 Dec 5;10(Suppl 5):128. doi: 10.1186/s12918-016-0371-3 (PMC5249043; doi:10.1186/s12918-016-0371-3)
Supplement: Additional file 1 — This file contains a long table of the drug indexes and names. (PDF 42 kb) [file 12918_2016_371_MOESM1_ESM.pdf]

Table 1: Drug indexes and names

Index	Drug Name	Index	Drug Name	Index	Drug Name
1	abacavir	2	acarbose	3	acebutolol
4	acetazolamide	5	acitretin	6	acyclovir
7	adapalene	8	adenosine	9	albendazole
10	alclometasone	11	alendronate	12	allopurinol
13	amantadine	14	amcinonide	15	amifostine
16	amikacin	17	amiloride	18	aminoglutethimide
19	aminophylline	20	amiodarone	21	amitriptyline
22	amlodipine	23	amoxapine	24	amoxicillin
25	ampicillin	26	amprenavir	27	anagrelide
28	anastrozole	29	apraclonidine	30	argatroban
31	aripiprazole	32	arsenic trioxide	33	aspirin
34	atenolol	35	atomoxetine	36	atorvastatin
37	atovaquone	38	atropine	39	azathioprine
40	azelaic acid	41	azelastine	42	azithromycin
43	aztreonam	44	bacitracin	45	baclofen
46	balsalazide	47	beclomethasone	48	benazepril
49	benztropine	50	betaxolol	51	bethanechol
52	bexarotene	53	bicalutamide	54	bimatoprost
55	biperiden	56	bisoprolol	57	bleomycin
58	bortezomib	59	bosentan	60	bretylium
61	brimonidine	62	brinzolamide	63	bromocriptine
64	budesonide	65	bumetanide	66	bupivacaine
67	bupropion	68	buspirone	69	cabergoline
70	caffeine	71	candesartan	72	capecitabine
73	captopril	74	carbachol	75	carbamazepine
76	carbinoxamine	77	carnitine	78	carteolol
79	carvedilol	80	cefaclor	81	cefadroxil
82	cefazolin	83	cefdinir	84	cefditoren
85	cefepime	86	cefixime	87	cefoperazone
88	cefotaxime	89	cefotetan	90	cefoxitin
91	cefpodoxime	92	cefprozil	93	ceftazidime
94	ceftizoxime	95	ceftriaxone	96	cefuroxime

97	celecoxib	98	cephalexin	99	cephradine
100	cetirizine	101	chloramphenicol	102	chloroquine
103	chlorothiazide	104	chlorpheniramine	105	chlorpromazine
106	chlorpropamide	107	chlorthalidone	108	chlorzoxazone
109	ciclesonide	110	cidofovir	111	cimetidine
112	cinoxacin	113	ciprofloxacin	114	citalopram
115	clemastine	116	clindamycin	117	clobetasol
118	clofazimine	119	clomipramine	120	clonidine
121	clotrimazole	122	clozapine	123	cromolyn
124	cyclobenzaprine	125	cycloserine	126	cyproheptadine
127	cysteamine	128	dacarbazine	129	danazol
130	dantrolene	131	dapsone	132	daunorubicin
133	delavirdine	134	demeclocycline	135	desflurane
136	desipramine	137	desloratadine	138	desoximetasone
139	dexamethasone	140	dexmedetomidine	141	dexrazoxane
142	diazoxide	143	diclofenac	144	dicloxacillin
145	dicyclomine	146	didanosine	147	diflunisal
148	digoxin	149	dihydroergotamine	150	diltiazem
151	diphenhydramine	152	dipivefrin	153	disopyramide
154	disulfiram	155	dobutamine	156	docetaxel
157	dofetilide	158	dolasetron	159	donepezil
160	dopamine	161	doxazosin	162	doxepin
163	doxorubicin	164	doxycycline	165	droperidol
166	dutasteride	167	echothiophate	168	econazole
169	efavirenz	170	emedastine	171	enalapril
172	enflurane	173	entacapone	174	epinephrine
175	eplerenone	176	eprosartan	177	ergotamine
178	ertapenem	179	erythromycin	180	esmolol
181	estradiol	182	estramustine	183	ethacrynic acid
184	ethambutol	185	ethionamide	186	ethosuximide
187	ethotoin	188	etodolac	189	etomidate
190	etoposide	191	exemestane	192	ezetimibe
193	famciclovir	194	famotidine	195	felodipine
196	fenofibrate	197	fenoldopam	198	fenoprofen
199	fexofenadine	200	finasteride	201	flavoxate

202	flecainide	203	fluconazole	204	fludarabine
205	fludrocortisone	206	flumazenil	207	flunisolide
208	fluocinonide	209	fluorometholone	210	fluoxetine
211	fluphenazine	212	flurbiprofen	213	flutamide
214	fluticasone	215	fluvastatin	216	fluvoxamine
217	formoterol	218	foscarnet	219	fosfomycin
220	fosinopril	221	fosphenytoin	222	fulvestrant
223	furosemide	224	gabapentin	225	galantamine
226	ganciclovir	227	gatifloxacin	228	gefitinib
229	gemcitabine	230	gemfibrozil	231	gemifloxacin
232	glimepiride	233	glipizide	234	glycopyrrolate
235	griseofulvin	236	guanethidine	237	guanfacine
238	halofantrine	239	haloperidol	240	halothane
241	hexachlorophene	242	hydralazine	243	hydrochlorothiazide
244	hydroflumethiazide	245	hydroxocobalamin	246	hydroxychloroquine
247	hydroxyurea	248	hydroxyzine	249	ibuprofen
250	ibutilide	251	idarubicin	252	iloprost
253	imatinib	254	imipramine	255	imiquimod
256	indapamide	257	indinavir	258	indomethacin
259	irbesartan	260	irinotecan	261	isocarboxazid
262	isoflurane	263	isoniazid	264	isoproterenol
265	isosorbide dinitrate	266	isradipine	267	itraconazole
268	ivermectin	269	kanamycin	270	ketamine
271	ketoconazole	272	ketoprofen	273	ketorolac
274	labetalol	275	lactulose	276	lamivudine
277	lamotrigine	278	lansoprazole	279	latanoprost
280	leflunomide	281	letrozole	282	leucovorin
283	levetiracetam	284	levobunolol	285	levocabastine
286	lidocaine	287	lincomycin	288	lindane
289	lisinopril	290	lithium	291	lomefloxacin
292	loperamide	293	loratadine	294	lorazepam
295	losartan	296	lovastatin	297	loxapine
298	malathion	299	maprotiline	300	mebendazole
301	mecamylamine	302	medroxyprogesterone	303	mefenamic acid
304	mefloquine	305	megestrol	306	meloxicam

307	meperidine	308	mephenytoin	309	mepivacaine
310	mercaptopurine	311	meropenem	312	mesoridazine
313	metaproterenol	314	metformin	315	methadone
316	methazolamide	317	methimazole	318	methotrexate
319	methyclothiazide	320	methyldopa	321	methylphenidate
322	methylprednisolone	323	metipranolol	324	metoclopramide
325	metolazone	326	metoprolol	327	metronidazole
328	mexiletine	329	miconazole	330	midodrine
331	mifepristone	332	miglitol	333	milrinone
334	minocycline	335	minoxidil	336	mirtazapine
337	mitotane	338	mitoxantrone	339	modafinil
340	moexipril	341	molindone	342	mometasone
343	montelukast	344	moricizine	345	morphine
346	moxifloxacin	347	mupirocin	348	mycophenolic acid
349	nabilone	350	nabumetone	351	nadolol
352	naftifine	353	nalbuphine	354	naloxone
355	naltrexone	356	naproxen	357	nateglinide
358	nelfinavir	359	neomycin	360	nevirapine
361	nicardipine	362	nicotine	363	nifedipine
364	nilutamide	365	nimodipine	366	nitric oxide
367	nitrofurantoin	368	nitroglycerin	369	nitroprusside
370	nizatidine	371	norepinephrine	372	norfloxacin
373	nortriptyline	374	ofloxacin	375	olanzapine
376	olopatadine	377	omeprazole	378	ondansetron
379	orlistat	380	orphenadrine	381	oxacillin
382	oxandrolone	383	oxaprozin	384	oxazepam
385	oxcarbazepine	386	oxiconazole	387	oxybutynin
388	oxytetracycline	389	paclitaxel	390	pamidronate
391	pantoprazole	392	paricalcitol	393	paromomycin
394	paroxetine	395	penbutolol	396	penciclovir
397	penicillin G	398	penicillin V	399	pentazocine
400	pentobarbital	401	pentoxifylline	402	perindopril
403	perphenazine	404	phenelzine	405	phenoxybenzamine
406	phentolamine	407	phenytoin	408	pilocarpine
409	pimecrolimus	410	pimozide	411	pindolol

412	pioglitazone	413	piperacillin	414	pirbuterol
415	piroxicam	416	polythiazide	417	pramipexole
418	pravastatin	419	prazosin	420	prednicarbate
421	prednisolone	422	prednisone	423	primaquine
424	primidone	425	probenecid	426	procainamide
427	procaine	428	prochlorperazine	429	procyclidine
430	progesterone	431	promethazine	432	propafenone
433	propantheline	434	propofol	435	propranolol
436	propylthiouracil	437	protriptyline	438	pyrazinamide
439	pyridostigmine	440	pyrimethamine	441	quetiapine
442	quinapril	443	quinidine	444	rabeprazole
445	raloxifene	446	ramipril	447	ranolazine
448	remifentanil	449	repaglinide	450	reserpine
451	ribavirin	452	rifabutin	453	rifapentine
454	riluzole	455	rimexolone	456	risedronate
457	risperidone	458	ritonavir	459	rivastigmine
460	ropinirole	461	ropivacaine	462	rosiglitazone
463	salmeterol	464	saquinavir	465	secobarbital
466	selegiline	467	sertraline	468	sildenafil
469	simvastatin	470	sotalol	471	spectinomycin
472	spironolactone	473	stavudine	474	streptomycin
475	sufentanil	476	sulfadiazine	477	sulfasalazine
478	sulfinpyrazone	479	sulfisoxazole	480	sulindac
481	sumatriptan	482	tacrolimus	483	tadalafil
484	tamoxifen	485	tamsulosin	486	tazarotene
487	telmisartan	488	temazepam	489	teniposide
490	terazosin	491	terbinafine	492	terbutaline
493	terconazole	494	testosterone	495	tetracycline
496	theophylline	497	thiabendazole	498	thioridazine
499	thyroxine	500	tiagabine	501	ticlopidine
502	tiludronate	503	timolol	504	tiotropium
505	tirofiban	506	tizanidine	507	tobramycin
508	tolazamide	509	tolazoline	510	tolbutamide
511	tolmetin	512	tolterodine	513	topiramate
514	topotecan	515	toremifene	516	tramadol

517	trandolapril	518	tranexamic acid	519	travoprost
520	trazodone	521	treprostinil	522	triamcinolone
523	triamterene	524	trifluoperazine	525	trihexyphenidyl
526	triiodothyronine	527	trimethoprim	528	trimetrexate
529	trimipramine	530	tropicamide	531	trovafloxacin
532	valacyclovir	533	valproic acid	534	valsartan
535	vancomycin	536	venlafaxine	537	verapamil
538	vinblastine	539	vincristine	540	vinorelbine
541	voriconazole	542	warfarin	543	zafirlukast
544	zalcitabine	545	zidovudine	546	ziprasidone
547	zolpidem	548	zonisamide		
